# Applications of the epidemiological modelling outputs for targeted mental health planning in conflict-affected populations: the Syria case-study

**DOI:** 10.1017/gmh.2016.4

**Published:** 2016-03-07

**Authors:** F. J. Charlson, Y. Y. Lee, S. Diminic, H. Whiteford

**Affiliations:** 1 School of Public Health, University of Queensland, Brisbane, Queensland, Australia; 2 Queensland Centre for Mental Health Research, Brisbane, Queensland, Australia; 3 Institute of Health Metrics and Evaluation, University of Washington, Seattle, Washington, USA

**Keywords:** Conflict, depression, global mental health, policy & systems, post-traumatic stress disorder, service planning

## Abstract

**Background:**

Epidemiological models are frequently utilised to ascertain disease prevalence in a population; however, these estimates can have wider practical applications for informing targeted scale-up and optimisation of mental health services. We explore potential applications for a conflict-affected population, Syria.

**Methods:**

We use prevalence estimates of major depression and post-traumatic stress disorder (PTSD) in conflict-affected populations as inputs for subsequent estimations. We use Global Burden of Disease (GBD) methodology to estimate years lived with a disability (YLDs) for depression and PTSD in Syrian populations. Human resource (HR) requirements to scale-up recommended packages of care for PTSD and depression in Syria over a 15-year period were modelled using the World Health Organisation mhGAP costing tool. Associated avertable burden was estimated using health benefit analyses.

**Results:**

The total number of cases of PTSD in Syria was estimated at approximately 2.2 million, and approximately 1.1 million for depression. An age-standardised major depression rate of 13.4 (95% UI 9.8–17.5) YLDs per 1000 Syrian population is estimated compared with the GBD 2010 global age-standardised YLD rate of 9.2 (95% UI 7.0–11.8). HR requirements to support a linear scale-up of services in Syria using the mhGAP costing tool demonstrates a steady increase from 0.3 FTE in at baseline to 7.6 FTE per 100 000 population after scale-up. Linear scale-up over 15 years could see 7–9% of disease burden being averted.

**Conclusion:**

Epidemiological estimates of mental disorders are key inputs into determining disease burden and guiding optimal mental health service delivery and can be used in target populations such as conflict-affected populations.

## Introduction

Epidemiological modelling to produce estimates of prevalence, incidence, mortality and remission has been used extensively in recent years, particularly by the various iterations of the Global Burden of Disease Studies (GBD). The purpose-designed software developed for GBD to conduct this modelling is able to produce epidemiological estimates disaggregated by age, sex, location and disorder which are subsequently used as inputs for burden of disease estimation – namely years lived with a disability (YLD), a component of the disability-adjusted life year (DALY) metric (Murray *et al*. 2012*a*). However, modelled epidemiological estimates can have wider practical applications when used as inputs for other epidemiological and health service estimations which are crucial for informing various aspects of mental health service planning.

Modelled epidemiological estimates have been applied (generally as data inputs) in work conducted by the World Health Organisation (WHO) and others. Examples include assessments of mental health workforce gaps (Bruckner *et al*. 2011) and development of models for scaling up recommended packages of care for priority mental and substance use disorders (Chisholm *et al*. 2007; Bruckner *et al*. 2011), and cost-effectiveness analyses of such mental health intervention packages (Chisholm *et al*. 2004; Gureje *et al*. 2007). In addition, epidemiological estimates can be incorporated as part of health benefit analyses to estimate the disease burden avertable by select intervention packages with known efficacy, applied at nominated target coverage rates (Andrews *et al*. 2000; Higashi *et al*. 2015).

Subpopulations, such as those affected by conflict, are not well represented by GBD estimates which are generally reflective of health loss at the country or regional level. Subsequently, models of mental health service requirements conducted to-date (which draw upon GBD estimates) are unlikely to represent the context or needs of conflict-affected populations. Predictive estimates of mental disorder prevalence in conflict-affected populations have been conducted before by Steel and colleagues demonstrating significantly elevated prevalence in these populations (Steel *et al*. 2009). These predictive prevalence estimates have been applied to estimate mental health service requirements for conflict-affected populations in Libya, a country affected by an intense conflict in 2011 (Charlson *et al*. 2012). However, key limitations in the methods of this predictive modelling were identified. Subsequently, a more recent predictive model, using updated data and significant advances in statistical methods, has derived revised estimates of major depression and post-traumatic stress disorder (PTSD) prevalence in conflict-affected populations (Charlson *et al*. 2015).

In this paper, we explore various ways in which epidemiological estimates of mental disorders can be applied to achieve targeted scale-up and optimisation of mental health services in a conflict-affected population – using Syria, a country currently experiencing ongoing conflict and health crisis (Coutts *et al*. 2013), as the case-example. By following GBD methodology and using subpopulation prevalence estimates, we re-estimate burden to more accurately reflect health loss attributable to major depression and PTSD in conflict-affected populations. Additionally, YLDs are projected forward for future planning of health services. The incorporation of age-and sex-specific epidemiological estimates into health economic models, such as the WHO's mhGAP tool (Chisholm *et al*. 2015), demonstrate how epidemiological estimates can be applied in estimating human resource (HR) requirements, and potential health gains for targeted scale-up of mental health services.

## Methods

### Epidemiological model estimates

Prevalence estimates of major depression and PTSD in conflict-affected populations, used as inputs in this study, have been previously developed using systematic review and Bayesian meta-regression methods. Methods have been described in full elsewhere (Charlson *et al*. 2015). In brief, a systematic review of the literature was conducted using PRISMA guidelines (Liberati *et al*. 2009) to collate epidemiological estimates of major depression and PTSD cases which met criteria for diagnosis as per the Diagnostic and Statistical Manual of Mental disorders (DSM) or the International Classification of Diseases (ICD) (World Health Organization, 1992; American Psychiatric Association, 2000). Electronic database searches were conducted using Medline (PubMed), PsychINFO, Embase, Published International Literature on Traumatic Stress (PILOTS), African Journal Online (AJOL), SciELO Public Health and ProQuest digital dissertations.

‘Conflict-affected’ countries were defined as meeting conditions set by the Uppsala Conflict Data Program (UCDP) database (Uppsala Conflict Data Program, 2012) and/or a level of 4 or 5 on the Political Terror Scale (PTS) (Political Terror Scale, 2011). To limit our populations to those either in conflict or within the 10-year post-conflict period, conflict status had to be met within the 10-year period prior to data collection. Studies were required to capture conflict-affected populations who remain within their country of origin or have been relocated to another neighbouring and/or non-Western country. Study samples not representative of the general population were excluded. Accepted diagnostic instruments were required to follow either ICD or DSM diagnostic criteria for major depression and PTSD.

DisMod-MR, a Bayesian meta-regression tool designed specifically for GBD 2010, was used to develop predictive estimates of age-specific prevalence in conflict-affected populations, whilst simultaneously adjusting estimates for heterogeneity between studies (Ferrari *et al*. 2013*a*, *b*). DisMod-MR generates estimates through a negative-binomial model that is fitted using a randomised Markov-Chain Monte Carlo algorithm (Vos *et al*. 2012*a*; Flaxman *et al*. 2013). These modelled estimates were age-standardised against the population of Syria for computations in this paper (United Nations, 2015).

### Prevalent cases

Given the widespread and protracted conflict taking place in Syria we assume that the entire population of Syria has been exposed to traumatic war-related events at some stage. Therefore, to derive estimated prevalent cases of major depression and PTSD, prevalence estimates were multiplied by the total population of Syria as documented by the UN World Population Data (United Nations, 2015). Prevalence estimates are drawn from a previously published meta-regression (Charlson *et al*. 2015) and age-standardised for the Syrian population distribution.

The proportion of PTSD cases comorbid with depression derived from general population and post conflict settings was set at 50% based on documented rates including a sample of Bosnian refugees living in Croatia (Mollica *et al*. 1999; Chapman *et al*. 2012).

### Burden of disease

As part of the Global Burden of Disease Study 2010 (GBD2010), burden of disease estimates are produced for 20 mental and substance use disorders (Whiteford *et al*. 2013). The core metric of health loss is the DALY which is made up of both mortality (yYears of life lost; YLL) and morbidity (YLD) components (Murray *et al*. 2012*b*). YLDs from a sequela are equal to the prevalent cases of the sequela multiplied by the disability weight for the health state associated with that sequela (Vos *et al*. 2012*b*).

Within a particular sequela, GBD considers there are several health states (e.g. mild, moderate and severe major depression). Each health state has its own disability-weight – a quantification of the severity of health loss associated with a health state on a scale from 0 to 1, when 0 is commensurate with perfect health and 1 is commensurate with death. In the GBD 2010, disability weights for health states are determined based on survey respondents representing the general public (Salomon *et al*. 2004). Comorbidity adjustments are applied to sequela-specific disability weights in the calculation of YLDs. YLDs for a disease or injury are the sum of the YLDs for each health state (e.g. mild, moderate and severe depression) associated with the disease or injury. More detailed information on GBD methodology has been found elsewhere (Murray *et al*. 2012*c*; Vos *et al*. 2012*b*; Whiteford *et al*. 2013).

We used GBD methodology and disability weights to estimate YLDs for major depression and PTSD in conflict-affected populations (Table 1). As GBD does not assess PTSD we have used disability-weights associated with overarching group of all anxiety disorders as proxy values. We used Ersatz (version 1.31, Brisbane, Australia; available at: http://www.epigear.com), an add-in that allows bootstrapping in Excel (Microsoft Corporation, Redmond, WA), to propagate uncertainty from the model input parameters through to the final YLD estimates (EpiGear International, 2012).

**Table 1.** GBD 2010 severity splits and disability-weights

Health state	Anxiety		MDD	
	Severity proportion, (95% UI)	Disability weight	Severity proportion, (95% UI)	Disability weight
Asymptomatic	30% (29–31)	0	14% (10–18)	0
Mild	38% (32–44)	0.03 (0.02–0.05)	59% (48–69)	0.16 (0.11–0.22)
Moderate	19% (15–23)	0.15 (0.10–0.21)	17% (12–21)	0.41 (0.28–0.55)
Severe	12% (8–17)	0.52 (0.37–0.68)	11% (4–20)	0.66 (0.47–0.82)
Average	–	0.11 (0.08–0.14)	–	0.23 (0.18–0.30)

### Mental health service requirements

Resource requirements to provide recommended packages of care for PTSD and depression were modelled using the WHO mhGAP costing tool (Chisholm *et al*. 2015). This tool utilises an Excel spreadsheet to calculate numbers of full time equivalent (FTE) staff, inpatient beds, bed days, and outpatient visits required to deliver basic packages of care for priority mental, neurological and substance abuse disorders. The mhGAP costing tool recommends several packages of care for each disorder, developed based on a systematic review of evidence and international consultation (World Health Organization, 2008). For depression, these include basic psychosocial treatment, advice and follow-up for moderate-to-severe cases; anti-depressant medication for moderate-to-severe cases; intensive psychosocial intervention for a subset of moderate-to-severe cases; and psychosocial care for perinatal depression. The package recommends that treatments for depression be delivered through a mix of primary health care, ancillary, outpatient and inpatient services. mhGAP does not include anxiety therefore we chose to apply the depression care packages and exclude inpatient treatment. The rationale for this is discussed later in the methods.

The calculation of resource requirements required data on the number of people eligible to receive a mhGAP intervention package. In our model, we assumed that these intervention packages were delivered to people older than 15 years who had a moderate–severe case of depression and/or PTSD. Epidemiological inputs for these calculations included: (a) the number of prevalent cases of PTSD, depression, and comorbid PTSD and depression in Syria, calculated as described earlier; and (b) the proportion of moderate and severe cases of depression (28%) and PTSD (30%), sourced from the GBD 2010 study (Table 1). Additional assumptions were made by the mhGAP tool to calculate the population eligible to receive the perinatal package of care (refer to online Table S1 in the appendix). The eligible population in this case was the proportion of all women in the population aged 15–44 years who gave birth during the year and had moderate–severe perinatal depression. We estimated a fertility rate of 11% based on United Nations Populations data (UNdata, 2012), that 15% had perinatal depression (O'hara & Swain, 1996; Fisher *et al*. 2012), and that the proportion of moderate–severe cases corresponded with the percentage for depression quoted above.

Each package of care includes a treatment coverage target (i.e. the proportion of prevalent cases to receive that package of care). Target coverage for 2030 was drawn from the default settings in the mhGAP costing tool (Table 2). A conservative baseline coverage rate of 1% was estimated from limited data available on current mental health service delivery in Syria (World Health Organisation, 2nd Quarter, 2014*a*, *b*). Although there are no clear signs of the conflict in Syria coming to an end we elected, for demonstration purposes, to use a hypothetical baseline year of services scale-up to be 2015.  To scale up the packages of care over 15 years from baseline to target coverage rates, two versions of the resourcing model were calculated. The first assumed an exponential scale up (i.e. small rate of scale-up initially with a more rapid rate of scale-up in the final years), while the second assumed a linear scale-up, where resources were increased at a constant rate over time.

**Table 2.** Recommended packages of care and coverage targets

Interventions	Target population	Coverage	
		Current (%)	Target (%)
*Depression*			
Basic psychosocial treatment, advice & follow-up	Moderate – severe cases of depression	1	30
Anti-depressant medication*	Moderate – severe cases of depression	1	30
Intensive psychosocial intervention	Moderate – severe cases of depression	1	10
Psychosocial care (home visits) for peri-natal depression	Moderate – severe cases of peri-natal depression	1	25
*PTSD*			
Basic psychosocial treatment, advice and follow-up	Moderate – severe cases of PTSD	1	30
Anti-depressant medication^a^	Moderate – severe cases of PTSD	1	30
Intensive psychosocial intervention	Moderate – severe cases of PTSD	1	10

a Anti-depressant medication = Fluoxetine, 20 mg tab; Amitriptyline, 50 mg tab.

Within each mhGAP package of care multiple treatment types may be included, such as primary care visits, ancillary care visits, and hospital inpatient and/or outpatient care. For each treatment type, the tool delineates the proportion of people in the care package who receive that treatment type, the number of visits or inpatient days required per person, and the number of minutes per visit. Further care package parameters are detailed in the online supplementary table.

Anxiety disorders are not included in the mhGAP costing tool. As evidence-based treatments for PTSD and major depression are similar (National Institute for Health and Care Excellence, 2009), the care packages for moderate to severe depression (excluding perinatal depression) were assigned for the treatment of moderate to severe PTSD. However, it was assumed that no inpatient treatment would be required for PTSD (National Institute for Health and Care Excellence, 2005). Prevalent cases of comorbid depression and PTSD were assigned treatment packages for depression, which include some inpatient treatment, as these cases are likely to require more intensive intervention.

The number of visits required for primary care, ancillary care and outpatient care in each care package were calculated as the product of the eligible population, the treatment coverage target, and the average number of visits per person. The number of bed days required for inpatient care in each care package was calculated as the product of the eligible population, the treatment coverage target, the percentage use and the number of bed days per person.

The numbers of visits or bed days were summed to provide a total for each treatment type and disorder. These were used to calculate the number of FTE staff required to deliver the recommended packages of care at the target coverage rate for each year. The mhGAP costing tool provides HR parameters for the following mental health provider types: psychiatrist, other physician/doctor, nurse, psychologist, other psychosocial worker and other provider/worker. For each provider type, the tool details the number of days worked per year, the average number of consultations per day for each community/outpatient treatment type, the average duration of a visit for each community/outpatient treatment type, and for inpatient care the number of staff per 25 bed ward. For each treatment type, the proportion of care delivered by each provider type is also detailed (see online supplementary table for full staffing parameters).

For each provider type, the number of FTE community/outpatient staff was calculated as the total number of visits in each setting multiplied by the proportion of care for that setting delivered by the relevant provider, divided by the number of consultations per year (number of consultations per day multiplied by the number of days worked per year). The number of inpatient beds was calculated as the number of bed days divided by 365 days per year, multiplied by 1.15 to assume 86% occupancy. For each provider type, the number of FTE inpatient staff was calculated as the number of beds divided by 25 beds per inpatient ward, multiplied by the number of that provider type per inpatient ward.

### Avertable burden with increasing treatment coverage

The following section outlines the steps used to calculate the burden of depression and PTSD that could be averted by scaling up mhGAP intervention packages in Syria between the years 2015–2030. A population model was constructed to calculate the change in DALY burden that would occur in a post-conflict country (Syria) under four intervention scenarios: (1) no treatment; (2) current coverage; (3) 30% target coverage; and (4) 100% target coverage. The ‘no treatment’ scenario calculated the DALYs arising under a hypothetical ‘partial null’ scenario where all existing mental health services are removed. The current coverage scenario calculated the DALYs that occur with the existing coverage rate (i.e. 1%). The third scenario analysed the DALYs arising from a gradual scale-up of intervention coverage from a baseline of 1% to a final target coverage of 30%. Finally, the fourth scenario analysed the DALYs that would occur if intervention packages were gradually scaled up to an ideal target coverage of 100% in the year 2030. The DALY burden in our model is reflective of the YLD burden given the low levels of case fatality attributable to depression and anxiety. As in the mhGAP analysis, we evaluated the effect of assuming a linear or exponential scale-up for each intervention scenario.

A multiple cohort Markov model was constructed in Excel to model the disease dynamics of several multi-age cohorts in the 2015 Syrian population (Habbema *et al*. 2010) (refer to online figure S1 in appendix). Our model was based on a previous disease model, DisMod 2 (Barendregt *et al*. 2003), which simulates how a population cohort moves between three health states (healthy, diseased and dead) on a year-to-year basis. Transitions between each health state correspond with defined epidemiological parameters (i.e. incidence, remission, case fatality and other cause mortality). Our model calculated the year-to-year transition of a multi-age cohort between each health state over a 15-year time horizon, which is in keeping with the time frame used in the mhGAP tool and is similar to the 10-year time period used in previous health economic studies (Edejer *et al*. 2012). The reference year of the analysis was 2015.

Multi-age cohorts were based on age groups contained in the mhGAP tool above 15 years of age – i.e. 15–29, 30–44, 45–59, 60–69, 70–79 and 80+ years. The mid-age population for each age cohort was used to model each cohort over the 15-year time horizon. For instance, the mid-age of the 15–29 year population is 22 years. So the population for the 15–29 year age cohort experienced the intervention over fifteen years using the mortality and disease parameters that applied to the ages 22–37 years. The next 30–44 year cohort had a mid-age of 37 years and was modelled between the ages 37–52 years and so on.

### Epidemiological input data

Input parameters were needed for the ages between 0 and 100 years to model the epidemiological experience of each age cohort over time. Population data were taken from UN population statistics. Mortality data for the 2015 Syrian population was obtained from GBD 2010.

DisMod 2 software was used to estimate a complete set of internally consistent disease parameters for PTSD and comorbid depression/PTSD in Syria. Prevalence data were obtained from the GBD 2010 study. A remission rate of 1.40 person-years was used for depression based on an average annual duration of 37.7 weeks. A remission rate of 0.17 person-years was used for PTSD based on the proportion of remitted cases obtained from a previous systematic review (Morina *et al*. 2014). The relative risk (RR) of mortality among depression cases was 1.9, based on a previous systematic review by Baxter 2011. The case fatality rate for PTSD was assumed to be zero at all ages. Entering the aforementioned input parameters into DisMod 2 allowed us to calculate internally consistent parameters on the incidence, prevalence, case fatality and remission for both depression and PTSD, respectively. Parameters were derived for 1-year age groups between the ages 0–100 years. These calculated parameters were, in turn, used as inputs for the multi-cohort Markov model.

### Intervention package details

We derived a set of intervention packages for the intervention scenario based on the intervention packages used in the mhGAP costing tool. Intervention packages, once again, targeted moderate to severe cases of depression and PTSD. The effect sizes for each intervention (expressed as the % change in remission and/or disability weight) were obtained from the previous work (Chisholm *et al*. 2000; World Health Organization, 2006; Chisholm & Saxena, 2012). The effectiveness of each intervention package was calculated by multiplying the efficacy of an intervention (e.g. % reduction in remission) by the total coverage (i.e. target population coverage multiplied by the scale-up coverage). The annual scale-up coverage was determined by the nominated intervention scenario. For example, scale-up coverage was constant over 15 years at 0% for the partial null and 1% for the current coverage scenarios. In the third and fourth intervention scenarios, the scale-up coverage (linear and exponential) increased from a baseline of 1% to a target coverage of 30 and 100%, respectively.  The combined effectiveness of each intervention package for each disorder was calculated by using the following equation (Edejer *et al*. 2012):
1.1
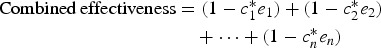

where *c* = coverage rate; *e* = effectiveness; and *n* = total number of interventions.

Two parallel life tables were constructed using the DisMod 2 equations to compare the effect of scaling up these intervention packages over a 15-year time horizon (i.e. intervention scenario) *v*. a no intervention scale-up scenario (i.e. comparator). This was done separately for PTSD and comorbid depression/PTSD to calculate the DALYs averted when interventions for these diseases are scaled up, respectively. Ersatz was used to implement the programming for the multi-cohort Markov model.

### Sensitivity analysis

Our baseline analysis did not apply a discount rate to account for the time preference of health benefits; where a benefit received now is worth more than a benefit received in the future. We thus conducted a sensitivity analysis to evaluate the impact of applying a 3% annual discount rate to the model.

## Results

### Prevalence

**Fig. 1.**
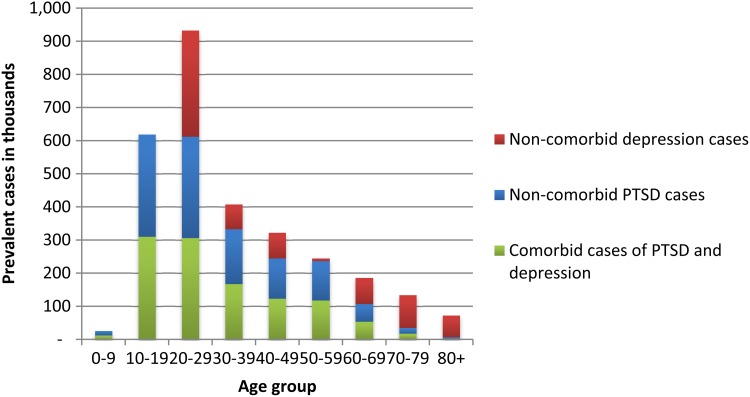
Age-specific prevalent cases, in millions, for PTSD and major depression in Syria, 2015.

The age-standardised prevalence of PTSD and major depression in Syria's conflict-affected populations is estimated at 12.9% (95% UI 6.9–22.9) and 7.6% (95% UI 5.1–10.9), respectively (Charlson *et al*. 2015). The total number of cases of PTSD in Syria was estimated at approximately 2.2 million, and at approximately 1.1 million for depression. When adjusting for high rates of comorbidity between the two disorders the combined number of prevalent cases was approximately 2.2 million. Both disorders demonstrate the highest number of prevalent cases in the adolescent to young adult age groups (Fig. 1). Based on population statistics available for Syria, this equates to approximately 1.3 million cases of depression and PTSD in the 20–29 year age-group alone or 2 million cases in ages 10–39.

### Burden of disease

Absolute YLD's for PTSD in Syria are estimated at approximately 250 000 [244 141 (95% UI 175 710–322 600)]. Age-standardised YLD rates per 1000 Syrian population are estimated at 13.1 (95% UI 9.4–17.3), compared to a GBD 2010 global age-standardised YLD rate per 1000 population (of ‘anxiety disorders’) of 3.9 (95% UI 5.4–2.7). Absolute YLD's for major depression in Syria are also estimated at approximately 250 000 [246 859 (95% UI 180 949–325 963)], or a rate of 13.4 (95% UI 9.8–17.5) YLDs per 1000 Syrian population. This is compared with the GBD 2010 global age-standardised YLD rate of 9.2 (95% UI 7.0–11.8).

**Fig. 2.**
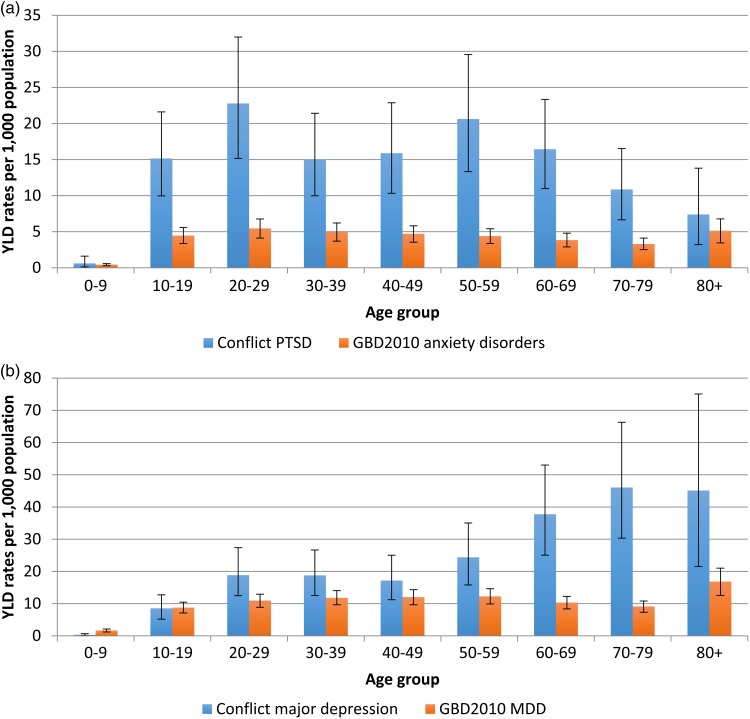
(a) Age-specific YLD rates per 1000 population (with 95% UI) for PTSD in Syria, 2015. (b) Age-specific YLD rates per 1000 population (with 95% UI) for major depression in Syria, 2015.

Age-specific YLD rates in conflict-affected populations demonstrate elevated and statistically significant differences to global YLD rates as estimated in GBD 2010. This is seen across the majority of age-groups despite large uncertainty intervals (UI) (Fig. 2).

### Mental health service requirements

#### FTE scale-up from 2015 to 2030

**Fig. 3.**
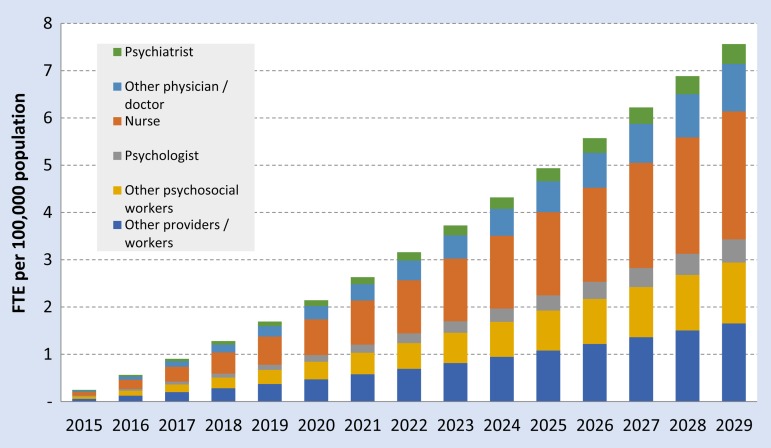
HR requirements for linear scale-up of anxiety and depression packages of care, in FTE per 100 000 population, 2015–2030.

Estimating HR requirements to support a linear scale-up of depression and anxiety health services in Syria using the mhGAP costing tool default settings demonstrates a steady increase from 0.3 FTE in 2015 to around 7.6 FTE per 100 000 population in 2029 (Fig. 3). This can be disaggregated by provider type with it being recommended that the vast majority of health workers are non-specialist. Using population projections of Syria we estimate approximately 50 mental health workers at present (baseline) with a target increase to 1600 by 2029 – consisting of approximately 570 nurses, 210 general physicians, 90 psychiatrists, 100 psychologists and 620 ‘other’ non-specialist providers.

Exponential scale-up models have the same estimations of HR requirements at baseline (2015) and 2029 but require less of an investment in the initial years of scale-up with the majority of the scale-up being achieved in the last few years. These models are available for reference in online Fig. 3*a, b* of the appendix.

Estimated scale-up demands on health services in terms of service contacts can be found in Table 3. The baseline estimates of service contacts total approximately 6000 outpatient visits, 37 000 primary care visits, and 78 000 ancillary care visits per annum. Total inpatient days for the baseline year are estimated at approximately 1000 days using only three dedicated inpatient beds. Inpatient services are reserved for cases of moderate–severe depression or comorbid depression/PTSD only as described in the methods. By the end of scale-up target service contacts will reach 2 million visits to each of primary care and ancillary care services; 300 000 outpatient service contacts and 44 000 inpatient days requiring 140 beds.

**Table 3.** Service contacts by year of linear scale-up for both PTSD and depression, 2015–2030

	2015	2020	2025	2029
PTSD				
Primary care visits	21 203	286 123	663 711	1 010 781
Ancillary care visits	26 802	134 210	287 022	425 905
Outpatient visits	3186	43 000	99 746	151 906
Inpatient days	–	−	–	–
Inpatient beds	–	–	–	–
Depression				
Primary care visits	16 120	221 709	537 155	844 998
Ancillary care visits	21 656	119 052	267 426	408 402
Outpatient visits	2423	33 320	80 727	126 991
Inpatient days	848	11 662	28 254	44 447
Inpatient beds	3	37	89	140
Total				
Primary care visits	37 323	507 832	1 200 866	1 855 780
Ancillary care visits	48 458	253 262	554 448	834 307
Outpatient visits	5609	76 320	180 473	278 897
Inpatient days	848	11 662	28 254	44 447
Inpatient beds	3	37	89	140

### Avertable burden with increasing treatment coverage

**Fig. 4.**
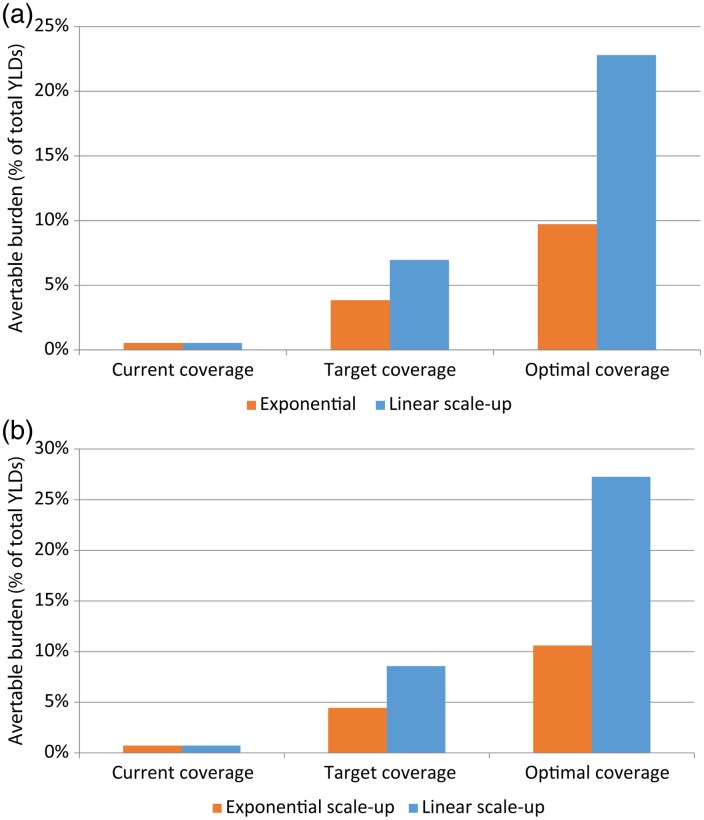
(a) Avertable burden (DALYs) of moderate-to-severe depression – three possible scenarios: (1) current treatment coverage; (2) scale up to 30% target coverage; (3) scale up to optimal (100% of moderate–severe cases) target coverage. (b) Avertable burden (DALYs) of moderate-to-severe PTSD – three possible scenarios: (1) current treatment coverage; (2) scale up to 30% target coverage; (3) scale up to optimal (100% of moderate–severe cases) target coverage.

Figures 4*a*, *b* demonstrate the health benefits that occur under a range of intervention scenarios based on three different coverage targets (current coverage, target coverage and 100% coverage) and two different models of scale-up (exponential and linear). Linear scale-up models make larger health gains over exponential scale-up models due to the more sustained growth throughout the scale-up period (in contrast to the growth being concentrated in the last few years). The modest coverage targets set in this analysis (10 to 30%, refer to Table 2) could see approximately 7–9% of the burden of disease attributable to moderate and severe cases of depression and PTSD averted (also refer to online Figure S4 of the appendix). Raising treatment coverage targets to 100% of moderate and severe cases would see approximately 23–27% of burden averted. Our results were robust even after applying a 3% discount rate (a difference of only 2% avertable burden – data not shown).

## Discussion

This paper demonstrates how epidemiological estimates of mental disorders can be applied to guide targeted scale-up and optimisation of mental health services in a post-conflict setting – Syria. Epidemiological estimates derived from the GBD Study and similar modelling exercises can provide a rich source of data for various components of informed mental health service planning and reform.

We estimate that around 2.2 million Syrian people are experiencing depression or PTSD, with half of those potentially suffering from both disorders. YLD rates for major depression in Syria are estimated at 13.4 (95% UI 9.8–17.5) YLDs per 1000 Syrian population, much greater than the GBD 2010 global age-standardised YLD rate of 9.2 per 100 000 (95% UI 7.0–11.8). This rate of burden is greater than the burden estimated for any other disease in GBD 2010 and highlights the mental health crisis likely present in the Syrian population.

In 2013 World Health Organization Syria initiated a program of providing mental health services using non-specialist health workers (World Health Organization, 2015). Implementation of mental health services has been successful in some areas of ongoing armed conflict (Ventevogel *et al*. 2012; Jordans *et al*. 2013). An eventual end to this conflict will present Syria with significant opportunities to rebuild and restructure its health and mental health services. What can be achieved by taking advantage of opportunities presented during phases of development have been highlighted in other countries with successful integration of mental health services (Raja *et al*. 2012) and mental health service reform (Makhashvili & van Voren, 2013).

It is important to note that the modelling of health service requirements presented in this paper is not a prescriptive assessment of mental health service scale-up in Syria. Any targets for scale-up would need to be considered within a range of contextual and feasibility factors (for example, it may not be realistic to achieve a target of 90 trained psychiatrists within a 15-year period) and adjusted accordingly. For the purposes of demonstrating what a scale-up of mental health services in a post-conflict period could potentially look like, we have presented two mental health service scale-up models – linear and exponential (refer to the online supplementary material). The linear model leads to greater health gains than the exponential model but requires a greater commitment of resources earlier in the scale-up period. The exponential scale-up assumption may be more realistic in the context of developing countries and post-conflict settings, which generally experience small incremental changes in the early years of program implementation.

HR requirements to support the 15-year scale-up of mental health services in Syria using the mhGAP costing tool demonstrates a steady increase from 0.3 FTE to 7.8 FTE per 100 000 population. It should be noted that this model only represents service requirements for depression and PTSD. Other mental disorders will require significant resources in addition to those presented in this paper. It is also vital to note the importance of embedding mental health services within primary healthcare (Patel *et al*. 2013) and task-shifting models which utilise non-specialist mental health workers as a feasible and effective mode of mental health services delivery in low-resource settings (Patel *et al*. 2009, 2011). WHO's mhGAP intervention guide for mental, neurological ad substance use disorders in low-resource settings is an essential resource for guiding the scale-up of mental health services (World Health Organization, 2008). Whilst PTSD is not a part of the core mhGAP intervention guide, it has been included in a subsequently published ‘mhGAP module Assessment Management of Conditions Specifically Related to Stress’ (World Health Organization, 2013).

The burden of disease that is avertable through the implementation of recommended treatment packages is perhaps modest compared with other diseases. However, these recommended treatment packages for mental disorders are very cost-effective and there is a strong economic argument for investing in mental health services in resource-constrained settings (Saxena *et al*. 2007; Buttorff *et al*. 2012). The scenario of 100% treatment coverage presented in our results is not a realistic goal but demonstrates the maximum avertable burden of disease through implementation of recommended packages of services.

The applications demonstrated in this paper are not exhaustive of what can be achieved with the availability and utilisation of additional data sources. For example, service provision costs would allow for full cost-effectiveness analyses, and service utilisation data can facilitate the exploration of global treatment gaps. The OneHealth software tool developed by the WHO is designed to inform national strategic health planning in low- and middle-income countries and is currently undergoing further development to enable full costing and service planning for mental health (http://www.who.int/choice/onehealthtool/en/).

### Limitations

The core limitations to the estimation methods presented in this paper stem from two main domains – (1) the epidemiological modelling; and (2) assumptions made in the modelling of service requirements. Perhaps the most important limitation of this current work is the omission of mental disorders other than PTSD and depression. As noted by the Inter-agency Standing Committee, ‘mental health and psychosocial problems in emergencies encompass far more than the experience of PTSD or disaster-induced depression. A selective focus on these two problems is inappropriate because it overlooks many other MHPSS problems in emergencies, as well as ignoring people's resources’ (Inter-Agency Standing Committee, 2007). As noted by Charlson *et al*. (2015), the available epidemiological literature for other mental disorders in conflict-affected populations is lacking. This leaves a significant gap in the modelling presented in this paper and limits our knowledge of how the mental health system should adapt and respond to address mental disorders collectively.

The limitations in deriving epidemiological estimates of depression and PTSD in conflict-affected populations using DisMod-MR software have been discussed elsewhere (Charlson *et al*. 2015). Further to this is the fact that no epidemiological data from Syrian populations was available for inclusion in the epidemiological model and all epidemiological estimates, including rates of comorbidity and proportions of moderate and severe cases, used in our analyses are drawn from other populations. The differences between Syrian and other conflict-affected populations cannot be understood until such a time that an epidemiological study of mental disorders in the general Syrian population can be undertaken.

Several assumptions were made in the modelling of service requirements and health benefits in this paper. Firstly, baseline coverage rates may not be reflective of the reality in Syria. Target coverage rates are also nominal and may over- or underestimate what can be achieved in a scale-up period. Effect sizes of recommended interventions may also not be representative of what can be expected in the Syrian population. All assumptions and inputs included in our models are guided by previously used models and/or published data; however, it is important to note that changes in any of these inputs can dramatically change derived findings presented in this paper.

As already noted, the service modelling presented in this paper is not a prescriptive assessment but rather a guide of what might be possible. Assumptions and targets for scale-up should be considered within a range of contextual and feasibility factors and adjusted accordingly. One such factor which has a large impact on modelling is the recent large reduction in population size of Syria, primarily due to refugees exiting the country. It might be reasonable to anticipate further considerable changes in population size as the crisis continues to evolve.

A further consideration when interpreting HR requirement estimations is that the mhGAP costing tool does not encompass non-clinical roles such as training and supervision. This is dealt with as an administrative overhead within the costing component of the tool and the costing exercise was considered outside the scope of this paper. The HR requirements needed to implement non-specialist delivered interventions as per the mhGAP-IG may therefore be underestimated in our results.

A final, but noteworthy, limitation with respect to scaling-up mental health services in a post-conflict environment is that ongoing instability will have serious and detrimental effects on any mental health system building efforts and also on the mental health of the population. Political and social stability is vital for successful health system building.

## Conclusion

Epidemiological estimates of mental disorders are key inputs into determining disease burden and guiding optimal mental health service delivery in target populations such as conflict-affected populations. Syria provides a topical case-study of how these estimates could be used to plan for mental health services in the wake of a devastating conflict and mental health crisis.

## Supplementary material

For supplementary material accompanying this paper visit http://dx.doi.org/10.1017/gmh.2016.4. click here to view supplementary material
