# Supplementary material for: Applications of the epidemiological modelling outputs for targeted mental health planning in conflict-affected populations: the Syria case-study
Source: Glob Ment Health (Camb). 2016 Mar 7;3:e8. doi: 10.1017/gmh.2016.4 (PMC5314753; doi:10.1017/gmh.2016.4)
Supplement: Supplementary file 1 [file S2054425116000042sup001.docx]

**Supplementary material**

Case fatality

Other mortality

Disease incidence

**Figure S1: Markov model of disease**

**Table S1: Assumptions and inputs**

| Human resource category |  | Average consultations / sessions per day | | | | Average duration of a visit (minutes) | | | Staffing proportion by health care setting | | | | | No. of staff per 25 bed inpatient ward |
| --- | --- | --- | --- | --- | --- | --- | --- | --- | --- | --- | --- | --- | --- | --- |
|  | Work days per year | Primary care | Ancillary care | Outpatient care | Day care | Primary care | Ancillary care | Outpatient care | Primary care | Ancillary care | Outpatient care | Day Care | Inpatient care |  |
| Psychiatrist | 240 | 6 | 4 | 8 | 2 | 15 | 20 | 20 | 2% | 5% | 10% | 5% | 10% | 1 |
| Other physician / doctor | 240 | 8 | 4 | 8 | 2 | 10 | 20 | 15 | 15% | 5% | 15% | 5% | 10% | 0.5 |
| Nurse | 240 | 10 | 8 | 10 | 2 | 10 | 30 | 15 | 50% | 30% | 30% | 50% | 50% | 4 |
| Psychologist | 240 | 6 | 4 | 6 | 2 | 45 | 45 | 45 | 3% | 5% | 10% | 10% | 10% | 0.5 |
| Other psychosocial workers | 240 | 8 | 6 | 8 | 2 | 20 | 30 | 30 | 10% | 25% | 20% | 20% | 10% | 0.5 |
| Other providers / workers | 240 | 8 | 8 | 8 | 2 | 15 | 20 | 15 | 20% | 30% | 15% | 10% | 10% | 1 |
| Total | 240 | - | - | - | - | - | - | - | 100% | 100% | 100% | 100% | 100% | 7.5 |

**Figure S2: HR requirements for linear scale-up of anxiety and depression packages of care, in FTE, 2015 to 2030**

**Figure S3a: HR requirements for exponential scale-up of anxiety and depression packages of care, in FTEs, 2015 to 2030**

**Figure S3b: HR requirements for exponential scale-up of anxiety and depression packages of care, in FTEs, 2015 to 2030**

|  |  |
| --- | --- |
| 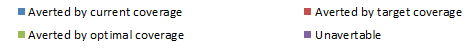 | |
| Depression avertable burden (linear scale-up) | PTSD avertable burden (linear scale-up) |

**Figure S4: Percentage of avertable burden by linear scale-up model**
